# Anthocyanins in Blueberries Grown in Hot Climate Exert Strong Antioxidant Activity and May Be Effective against Urinary Tract Bacteria

**DOI:** 10.3390/antiox9060478

**Published:** 2020-06-02

**Authors:** Ana B. Cerezo, Giorgiana M. Cătunescu, Mercedes Martínez-Pais González, Ruth Hornedo-Ortega, Carmen R. Pop, Crina Claudia Rusu, Flore Chirilă, Ancuța M. Rotar, M. Carmen Garcia-Parrilla, Ana M. Troncoso

**Affiliations:** 1 Departamento de Nutrición y Bromatología, Toxicología y Medicina Legal, Facultad de Farmacia, Universidad de Sevilla, C/Profesor García González 2, 41012 Sevilla, Spain; acerezo@us.es (A.B.C.); marmargpub@gmail.com (M.M.-P.G.); mcparrilla@us.es (M.C.G.-P.); 2 Department of Technical and Soil Sciences, Faculty of Agriculture, University of Agricultural Sciences and Veterinary Medicine Cluj-Napoca, Calea Mănăștur 3-5, 400372 Cluj-Napoca, Romania; 3 Axe Molécules d’Intérêt Biologique (MIB), Unité de Recherche Œnologie, EA4577, USC 1366 Institut National de la Recherche Agronomique (INRA), Institut des Sciences de la Vigne et du Vin (ISVV), Université de Bordeaux, 33882 Bordeaux, France; ruth.hornedo-ortega@u-bordeaux.fr; 4 Department of Food Science, Faculty of Food Science and Technology, University of Agricultural Sciences and Veterinary Medicine Cluj-Napoca, Calea Mănăștur 3-5, 400372 Cluj-Napoca, Romania; carmen-rodica.pop@usamvcluj.ro (C.R.P.); anca.rotar@usamvcluj.ro (A.M.R.); 5 Department of Nephrology, University of Medicine and Pharmacy “Iuliu Hatieganu”, Victor Babeş 8, 400012 Cluj-Napoca, Romania; claudia.rusu@umfcluj.ro; 6 Department of Clinical and Paraclinical Sciences, Faculty of Veterinary Medicine, University of Agricultural Sciences and Veterinary Medicine Cluj-Napoca, Calea Mănăștur 3-5, 400372 Cluj-Napoca, Romania; flore.chirila@usamvcluj.ro

**Keywords:** antibacterial activity, *Escherichia coli*, *Klebsiella pneumoniae*, *Pseudomonas aeruginosa*, *Providencia stuartii*, *Micrococcus*, cyanidin-3-rutinoside, ORAC, UHPLC-MS/MS Orbitrap

## Abstract

Anthocyanins are extensively studied for their health-related properties, including antibacterial activity against urinary tract infections (UTI). Among common fruits, blueberries, with their remarkable antioxidant capacity, are one of the richest sources. Anthocyanin-rich extracts were obtained from four varieties: Snowchaser, Star, Stella Blue and Cristina Blue, grown in the hot climate of Southern Spain. Their total anthocyanins contents (TAC) were determined spectrophotometrically, and the anthocyanin profile by ultra high performance liquid chromatography—tandem mass spectrometer (UHPLC-MS/MS). Their antioxidant activity was assessed by oxygen radical absorbance capacity (ORAC) assay, while antibacterial activity against strains isolated from UTI patients was assessed in vitro, helping to select the varieties with the highest bioactive potential. Star showed the highest TAC and antioxidant activity (1663 ± 159 mg of cyanidin-3-*O*-glucoside (cy-3-*O*-glu) equivalents/100 g fresh weight (FW), 6345 ± 601 μmol Trolox equivalents (TE)/100 g FW, respectively), followed by Cristina Blue, Stella Blue and Snowchaser. As far as we know, this is the first time that cyanidin-3-rutinoside has been identified in blueberries. The extracts inhibited all the tested strains, MICs ranging from 0.4 mg/mL (for Stella Blue extract against UTI *P. aeruginosa*) to 9.5 mg/mL (for all extracts against UTI *K. pneumoniae* ssp. *pneumoniae*). This is the first study that assessed in vitro the antibacterial activity of blueberries against *Klebsiella pneumoniae*, *Providencia stuartii* and *Micrococcus* spp. strains isolated from UTI.

## 1. Introduction

Berries contain high amounts of polyphenols, including flavonoids, and are widely consumed globally. Due to their healthful properties in a broad number of biological functions [1], anthocyanin pigments, considered to be the most abundant flavonoids in berries, have been extensively studied. There are currently more than 600 different anthocyanins described in the plant kingdom [2,3,4,5,6,7]. Their reported healthy effects are mainly related to their antioxidant and anti-inflammatory activity in the prevention of some metabolic disorders [8]. Recent studies have shown that an increase in anthocyanin dietary intake (from 3 to 24 mg/day) is significantly associated with a 12–32% [9,10,11] reduction in the risk of coronary heart disease. Cassidy et al. [9] have shown that, for every 15 mg increase in anthocyanin consumption, the relative risk of myocardial infarction decreases by 17%. Notably, berries are the main food source of anthocyanins in Europe (11%) [12]. The consumption of three servings per week (approximately 225 g) of strawberries or blueberries has been associated with a significant decrease in the risk of myocardial infarction [9] and overall cardiovascular risk [13]. Additionally, berries of the *Vaccinium* genus have shown potential for use in urinary tract infections (UTI). Anthocyanins extracted from cranberries are widely known as an adjuvant in preventing or treating UTI [14,15,16,17,18]. Blueberries (*Vaccinium corymbosum* L.) present a significantly higher concentration (32–407 mg/100 g fresh weight (FW)) and diversity of anthocyanins (10–23) compared to other berry fruits, such as strawberries, grapes and raspberries (27–48 mg/100 g FW; seven to seventeen different compounds) [19,20,21,22,23,24,25,26,27]. Among common fruits, blueberries, therefore, are one of the richest sources of anthocyanins, making them extremely interesting fruits in terms of bioactive potential, with a remarkable antioxidant capacity [28,29]. 

It is not surprising that blueberry cultivation is currently booming. Southern Spain is the leading blueberry producer in Europe and fourth in the world. Blueberry production is rapidly increasing because of its excellent productivity, adaptability to different environments and pest resistance [30]. It is worth noting that both the anthocyanin profile and their respective concentrations vary significantly among the different varieties, growing areas and also intra- and inter-harvests [20,31,32,33,34]. Most of the varieties grown in Andalusia, a region located in Southern Spain, Cielo, Katiblue, Rockinoee, Star, Stella Blue, Terrapin, Emerald, Jewel, Snowchaser, Rocío, etc., have been adapted to the warm climate of southern Europe, taking advantage of an earlier January–June maturing season than the rest of Europe. 

To date, most studies carried out on the anthocyanin profile of blueberries have focused on varieties grown in countries such as the USA, Chile, Germany, Italy or Canada (*V. corymbosum* var. Nui, Darrow, Reka, Puru, Bluegold, Berkeley, Legacy, Sampson, Elliott, Pamlico) [20,22,25,31,35,36,37]. The genotype (variety) has a significant impact on the concentration and anthocyanin profile of blueberries, determining not only their overall concentration but also the major anthocyanins [20,21,31,32,33,34,38]. Those anthocyanins most characteristic of blueberries are glycosides (glucosides, galactosides and arabinosides) and, in a smaller proportion, acetylated derivatives of cyanidin, delphinidin, malvidin, peonidin and petunidin. The varieties that have shown the highest concentration of total anthocyanins were Elliott and Pamlico (407 and 384 mg/100 g FW, respectively) [22,38].

Furthermore, it has been shown that the cultivation area also plays a key role in both the anthocyanin profile and total concentration. For the Legacy variety, total anthocyanins are significantly higher when cultivated in the warm climate of North Carolina (USA) (261 mg/100 g FW), as compared with the continental climate of North-East Romania (189 mg/100 g FW) [31,38]. Blueberry cultivation enables fruits to be collected as many as eight times from the same bush per season. Furthermore, the concentration and profile also vary significantly within and between harvests, due to changes in environmental conditions such as temperature and rainfall. Therefore, anthocyanin concentrations are higher in fruits collected in hotter (30–33 °C) months and years with a lower rainfall (7–47 mm) [20]. In conclusion, a warm climate and high temperatures yield fruits with a higher anthocyanin concentration.

The United States is the world’s largest producer of blueberries [39]. More recently, Andalusia, Spain, has become an important producer and is at present the largest producer in Europe (51.569 tonnes in 2019) [40]. Nevertheless, only one study has evaluated the anthocyanin profile of three different Andalusian varieties: Rocío and two other experimental varieties (V2 and V3). They contained glycosides and acetylated derivatives of delphinidin, petunidin, cyanidin and malvidin (not peonidin), with malvidin 3-hexoside being the major anthocyanin [41]. The authors also demonstrated that the bush’s genotype has a significant effect on the anthocyanin profile, and that their concentrations vary between harvests. It is worth noting that some varieties currently under cultivation in Andalusia, such as Snowchaser, Emerald and Jewel, accumulated total anthocyanins ranging from 63–101 mg/100 g in other blueberry-producing regions [42,43]. 

Many studies have reported the antibacterial activity of anthocyanins extracted from blueberries against various Gram-positive (*Listeria monocytogenes*, *Staphylococcus aureus* and *Clostridium perfringens*) and Gram-negative (*Salmonella enterica*, *E. coli* and *Campylobacter* spp.) foodborne pathogens [44,45,46,47,48,49,50,51,52,53,54]. However, many of these studies were performed with complex extracts, obtained using different methods. It is not entirely clear which particular compounds are responsible for the antibacterial activity observed [55].

Similar to cranberries, blueberries are rich in anthocyanins, but their efficacy as an adjuvant in preventing or treating UTI is still in doubt and unclear [18,56]. Only a few in vitro studies have tested their effect against uropathogenic *E. coli* strains isolated from the urine of human patients diagnosed with UTI, and only one against *Pseudomonas aeruginosa* [50,57,58]. Although these findings appear promising, researchers have yet to find the array of susceptible pathogens. It is, therefore, currently of interest to evaluate the antibacterial activity of blueberry anthocyanins, not only against foodborne pathogens, but also against bacteria strains associated with UTI infections. Moreover, similar promising results were obtained in the case of other berries different to cranberries such as *Aronia melanocarpa* [59]. 

Anthocyanins extracts might be attractive adjuvants and/or alternatives to synthetic antibiotics, because they contain dynamic combinations of bioactive phytochemicals that might combat resistance on various complementary levels [47]. Current results show that complex mixtures of blueberry extracts show a better antibacterial efficacy against *Salmonella* and *Campylobacter* compared to individual compounds [60], most likely because of the synergy among the phytochemicals [60,61,62]. Thus, investigating complex anthocyanin mixtures instead of the purified compounds would appear justifiable.

Since substantial differences have been found in terms of anthocyanins concentration and diversity in blueberries grown in countries with different climatic conditions, their bioactive properties may thus vary, due to changes in their anthocyanin profiles. In order, therefore, to select those varieties that show an optimum anthocyanin profile and potent bioactive properties, identifying and quantifying the anthocyanins of blueberries grown in Andalusia is of great importance.

The aim of the present study is to evaluate the total anthocyanins content (TAC) and the anthocyanins profile of four blueberry varieties: Snowchaser, Star, Stella Blue and Cristina Blue, which are grown in Andalusia, in order to determine which variety presents the highest TAC, and to discriminate them based on their anthocyanins composition. Simultaneously, we intend to assess the antioxidant activity and the in vitro antibacterial activity of the extracted anthocyanins against standard pathogenic strains and bacteria isolated from patients suffering from UTI. This would enable those varieties with the greatest bioactive potential to be selected. 

To the best of our knowledge, not only is this the first attempt to evaluate the TAC and anthocyanins profile of Snowchaser, Star, Stella Blue and Cristina Blue grown in Andalusia, but it is also the first evaluation of the in vitro effect of blueberry anthocyanins on potential uropathogenic bacteria strains, such as: *Klebsiella pneumoniae*, *Providencia stuartii* and *Micrococcus* spp.

## 2. Materials and Methods 

### 2.1. Reagents

Amberlite XAD7HP, AAPH (2,2′-diazo-bis-amidinepropane-dihydrochloride) and Trolox (6-hydroxy-2,5,7,8-tetramethylchroman-2-carboxylic) were purchased from Sigma (Munich, Germany). Fluorescein was provided by Fluka. Methanol for liquid chromatography and acetic acid 99.8% were purchased from Merck (Munich, Germany) and VWR CHEMICALS (Radnor, PA, United States), respectively. Malvidin-3-glucoside, cyanidin-3-glucoside, cyanidin-3-galactoside, peonidin-3-glucoside and delphinidin-3-glucoside were purchased from Extrasynthese (Genay, France). 

### 2.2. Samples

Four different varieties of highbush blueberry (*Vaccinum corymbosum*) were analysed: Snowchaser, Star, Stella Blue and Cristina Blue. They were all grown in the hot climate of Huelva, Southern Spain, in the towns of Palos de la Frontera and Almonte. Blueberries at commercial ripening were sampled from February to March 2019, depending on when the harvest of each variety commenced.

### 2.3. Extraction of Anthocyanin Fraction

Once collected, the whole blueberries (200 g FW) were immediately frozen at −80 °C for at least 24 h and subsequently freeze-dried. The resultant dry samples were independently mixed with 200 mL of acidified methanol (0.5% acetic acid) using a homogeniser [20,21,38], before being centrifuged at 3452 g for 10 min at 20 °C, and the supernatant fraction then being collected. Re-extraction was performed until the pellet was colourless. The supernatant of each variety was filtered, concentrated under vacuum at 35 °C to remove methanol, and then diluted 1:1 with water. The purification of the anthocyanins fraction of each variety was performed as previously described in the literature [6,20,21,23,63], using an Amberlite XAD-7 column (30 × 1.5 cm) previously activated with methanol, and then 300 mL of water. Samples were loaded onto the column and cleaned with 450 mL of water, in order to remove free sugars, pectin, and organic acid, among other polar compounds. The anthocyanin fraction was eluted with methanol/acetic acid solution (19:1, v/v) at 1 drop/s flow, concentrated under vacuum, frozen at −80 °C and freeze-dried to obtain the anthocyanin extract (Table 1).

### 2.4. Total Anthocyanin Content (TAC)

Total monomeric anthocyanin content was determined by the pH differential method [64]. Solutions used were potassium chloride buffer (KCl) at pH = 1.0 (0.025 M) and sodium acetate buffer (C_2_H_3_NaO_2_) at pH = 4.5 (0.8 M). The anthocyanin extract of each variety was prepared twice: once with potassium chloride buffer (pH 1.0) and then with sodium acetate buffer (pH 4.5). They were settled for 15 min before their absorbances were measured in the spectrophotometer. Absorbance (A) was measured at 500 nm and 700 nm for each sample in both buffers. Samples were analysed in triplicate (duplicates of three different days). The following formulae were applied to estimate the total anthocyanin content:

A = (A_500_ − A_700_) pH 1.0 − (A_500_ − A_700_) pH 4.5 (1)

TAC (mg/100g FW) = (A × MW × 1000)/(ε × 1)(2)

where MW is the molecular weight of cyanidin-3-*O*-glucoside (cy-3-*O*-glu) (MW = 449.2 g/moL), and ε its molar absorptivity (26,900 L cm^−1^ mol^−1^). Total anthocyanin content (TAC) is expressed as mg cy-3-*O*-gluc/100 g of FW.

### 2.5. Oxygen Radical Absorbance Capacity (ORAC)

The antioxidant capacity was measured by oxygen radical absorbance capacity (ORAC), as described in Ou et al. [65]. A total of 50 μL of the extracts or Trolox (0.5–15 μM) were mixed with 100 μL of fluorescein (90 nM) and 50 μL AAPH (15 nM) in black 96-well plate. Phosphate buffer and fluorescein controls were also included. A total of 50 μL of phosphate buffer, 100 μL of fluorescein and 50 μL of AAPH were used as blank. The analysis was performed at 37 °C. Fluorescence at excitation and emission wavelengths of 485 nm and 528 nm, respectively, was recorded every 5 min for 60 min using a multi-detection microplate reader (Synergy HT, Biotek, Winooski, VT, United States). Samples were analysed in triplicate (triplicates on three different days).

The ORAC values were calculated using the differences between the blank and the sample areas under the fluorescein decay curve. Results are expressed as μmol Trolox equivalents (TE)/100 g of FW.

### 2.6. Identification of Anthocyanins: UHPLC-MS/MS Orbitrap

Identification of the blueberries’ anthocyanins was performed by a UHPLC-MS/MS (ultra-high- performance liquid chromatography) coupled to a hybrid quadrupole-orbitrap mass spectrometry system (Qexactive, Thermo Fisher, Waltham, Massachusetts, United States) with electrospray ionisation (HESI-II). The analytical conditions were previously described by Hornedo-Ortega et al. [63]. Separation was carried out using a reverse-phase ZORBAX SB-C18 rapid resolution HD (2.1 × 100 mm, 1.8 μm) column (Agilent, Santa Clara, CA, United States). The injected volume was 1 μL (extract dissolved in mobile phase A) and the flow was 0.4 mL/min. The mobile phase (A: water/formic acid 95:5, v/v; B: acetonitrile/formic acid 95:5, v/v) gradient was as follows: 0–2 min 5% B, 2–12 min from 5% to 100% B, 12–13 min from 100 to 5% B, and 5% B for 15 min. Analyses were carried out using full MS scan from 100–1500 *m/z*, and high collision energy dissociation (HCD). The MS/MS parameters were as follows: positive ionisation mode, resolution of 35000, 20 eV per cell, 3.5 kV of voltage, 50 V in the lens of the channel, capillary temperature 320 °C, 12.5 and 50 (arbitrary units) flux of the auxiliary gas (N_2_) and gas boosting. Xcalibur Software (version 3.0.63, Waltham, Massachusetts, United States) was used to analyse the data. Identification was performed according to their accurate molecular mass, molecular formula, calculated mass, characteristic fragmentation and retention time. The following anthocyanin standards were used for identification purposes: malvidin-3-glucoside, cyanidin-3-glucoside, cyanidin-3-galactoside, peonidin-3-glucoside and delphinidin-3-glucoside.

### 2.7. Bacterial Strains

#### 2.7.1. Standard Strains

Standard strains, *Escherichia coli* ATCC (American Type Culture Collection) 25922, *Salmonella* Enteritidis ATCC 13076, *Listeria monocytogenes* ATCC 19114 were tested as controls. They were grown in a test tube containing 10 mL sterile nutrient broth (Oxoid Ltd., Basingstoke, Hampshire, England) at 37 °C for 24 h. A loopful of inoculum was transferred onto selective media: TBX agar for *E. coli*, XLD agar for *Salmonella* Enteritidis (Oxoid Ltd., Basingstoke, Hampshire, England) and Palcam agar base supplemented with Listeria Palcam antimicrobic supplement (Oxoid Ltd., Basingstoke, Hampshire, England) for *Listeria monocytogenes*. Plates were incubated for 24 h at 37 °C. Bacterial morphology was confirmed by optical microscopy.

#### 2.7.2. Uropathogenic Bacteria Isolated from UTI Patients 

Urine was collected from 6 human patients who had given their written consent, in accordance with the ethics protocol of the collecting hospital, the County Emergency Hospital Cluj-Napoca, Romania (SCJU). The inclusion criteria were age above 18 years and the presence of clear clinical signs of UTI: hypogastric pain or/and dysuria or/and pollakiuria or/and disturbed urine or/and renal colic [66]. The exclusion criteria were antibiotic treatments 48 h previous to sampling and absence of leucocytes and/or of nitrites on dipstick analysis [66]. The selected patients were instructed on how to collect the samples. Their clean catch mid-stream urine samples were collected using the provided wide mouth 50 mL sterile universal containers, according to the current widely accepted Danish recommendations [66]. The secure-closed containers, each containing at least 20 mL of urine, were sent for microbiology testing. Each urine specimen was cultured within 30 min of sample collection as follows: 50 μL of urine was vortexed and then inoculated on glucose agar plates using a sterile loop and incubated in aerobic conditions at 37 °C for 18–24 h. For the plates where bacterial growth was observed, the bacteria were identified by the conventional morphological and standard culture-based methods and by biochemical characteristics using the Vitek 2 system [67,68]. The colonies’ genus and species were identified by microscopic examination of the Gram-stained smear to assess the morphology of the cells’ shape, size and the presence of pigments. The strains were also biochemically characterised using the Vitek 2 system, according to the manufacturer’s instructions. In the end, 6 bacteria of UTI importance were isolated and identified, 4 Gram-negative: *Escherichia coli* β-Haemolytic, *Providencia stuartii*, *Klebsiella pneumoniae* ssp. *pneumoniae*, *Pseudomonas aeruginosa* and 1 Gram-positive *Micrococcus* spp.

#### 2.7.3. Preparation of Bacterial Strains

Several colonies of standard and UTI bacteria cultivated on Mueller-Hinton agar (Oxoid Ltd., Basingstoke, Hampshire, England) were transferred into sterile saline solution (8.5 g/L) and adjusted to match the turbidity of McFarland 0.5 standard (1.5 × 10^8^ CFU/mL). Then, a bacterial suspension of 1.5 × 10^6^ CFU/mL was prepared for addition to each microplate well.

### 2.8. Determination of the Minimum Inhibitory Concentration (MIC)

The MIC was determined using the resazurin microtiter plate-based antibacterial assay [69,70]. Fresh stock methanolic solutions of the blueberry anthocyanin-rich extracts were prepared each experimental day at concentration of 20 mg/mL of 70% methanol. One hundred microliters of sterile nutrient broth (Oxoid Ltd., Basingstoke, Hampshire, England) were added in the wells of a 96-well microplate. Then 100 µL of stock solutions were added in the first wells of each row and serial 11-fold dilutions were performed in the subsequent wells of each row by transferring 100 µL from well to well. The surplus 100 µL in the last well of the row was discarded. Then, 10 µL of inoculum (1.5 × 10^6^ CFU/mL) was added to all wells. The actual tested concentrations of the methanolic solutions of the anthocyanin-rich extracts were: 9.520 mg/mL; 4.530 mg/mL; 2.182 mg/mL; 1.038 mg/mL; 0.494 mg/mL; 0.235 mg/mL; 0.112 mg/mL; 0.053 mg/mL; 0.025 mg/mL; 0.012 mg/mL; 0.006 mg/mL; 0.003 mg/mL. Gentamicin (0.04 mg/mL in saline solution) was the positive control, and 70% methanol was the negative control.

The microplates were incubated for 20–22 h at 37 °C and, then 20 µL of 0.2 mg/mL resazurin aqueous solution was added to all wells. The microplates were incubated for another 2 h at 37 °C. After this period of incubation, resazurin (a blue non-fluorescent dye) was oxidised to resorufin (fluorescent pink) wherever the wells contained viable bacterial cells. The concentration in last blue well on each row was considered the lowest that completely inhibited bacterial growth, thus the MIC. Three replicates were performed for each bacterium and each stock methanolic solutions.

### 2.9. Determination of the Minimum Bactericidal Concentration (MBC)

MBC was determined by plating 10 μL from the last 4 blue wells on each row (the 4 lowest concentrations that showed inhibition of bacterial growth) in the MIC testing on Mueller-Hinton solid culture medium (Oxoid Ltd., Basingstoke, Hampshire, England) [69]. The plates were incubated for 24 h at 37 °C. The lowest concentration that prevented bacteria growth (no colonies on the plate) was considered to be the MBC. Three different biological replicates were performed for each plate and all experiments were performed in duplicate.

### 2.10. Statistical Analysis

The results were statistically analysed using Graphpad Prism software (GraphPad Software, Inc., San Diego, CA, USA) and XLSTAT software (Addinsoft, New York, New York, USA, XLSTAT 2020.1.3.65245) [71]. Student’s t-test was used to analyse the obtained data from TAC and ORAC, with differences at *p* < 0.01 considered significant. A one-way ANOVA (*p* < 0.05) within samples was used to compare the 4 blueberry varieties and their antibacterial effect against the tested bacteria. Fisher pairwise comparisons (LSD, *p* < 0.05) were made whenever ANOVA indicated a significant difference. Pearson correlational analyses were performed to examine the TAC/ORAC relationship and antibacterial activity. Whenever a correlation was confirmed, a linear regression was performed with a 95% confidence interval, a tolerance of 0.0001 and a model selection based on best model by *R^2^*, to establish and quantify the effect of TAC and antioxidant activity (ORAC) on the antibacterial activity.

## 3. Results and Discussion

### 3.1. Total Anthocyanins Content (TAC)

**Figure 1:**
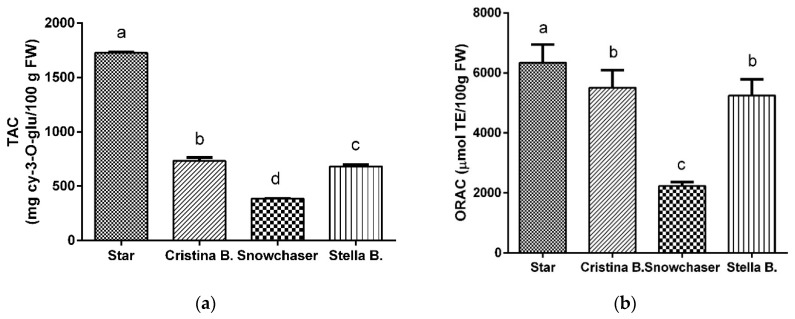
Total anthocyanin concentration (**a**) and antioxidant activity (**b**) of the different blueberry varieties. Total anthocyanin content (TAC) results are expressed as mg of cy-3-*O*-gluc/100 g fresh weight (FW) and oxygen radical absorbance capacity (ORAC) values as µmol of Trolox equivalents/100 g FW. Values represent mean ± SD of three replicates (*n* = 3). Different letters mean significant differences between the four varieties at a level *p* < 0.01 (Student’s *t*-test).

Figure 1 shows that Star was the variety with the significant highest anthocyanin concentration (1663 ± 159 mg cy-3-*O*-glu/100 g FW), followed by Cristina Blue (733.4 ± 30.9 mg cy-3-*O*-glu/100 g FW), Stella Blue (682.6 ± 14.7 mg cy-3-*O*-glu/100 g FW) and Snowchaser (384.8 ± 4.4 mg cy-3-*O*-glu/100 g FW). The TAC of Snowchaser has previously been reported for fruits cultivated in the South of Brazil (62.36 mg cy-3-*O*-glu/100g FW) [42]. Despite the fact that both the South of Brazil and Spain share a hot climate, total Snowchaser anthocyanin was six times higher when cultivated in Spain. TAC values ranging 31.54–406.9 mg cy-3-*O*-glu/100 g FW have been reported for Agropaine, Arlen, Berkley, Blomidom, Bluecrop, Bluegold, Bluejay, Brigitta, Chipava, Darrow, Duke, Elliott, Hannah’s Choice, Legacy, Lenoir, Nelson, North Country, Northblue, Northland, Nui, O’Neal, Pamlico, Sampson, Toro, Jersey, Croatan, Rancocas and Rubel varieties [22,25,31,36,38,72,73]. All of the above were grown in the USA, Romania, Slovenia, Korea and Chile, but not in Spain. Our results show that Star, Cristina Blue and Stella Blue varieties cultivated in Southern Spain exhibited higher TAC values than previously published. Interestingly, Star presents four times higher anthocyanin content than the richest variety in anthocyanins (Elliott) so far reported [22].

Our results show that Star, Cristina Blue, Stella Blue and Snowchaser blueberries contain higher anthocyanin concentrations compared with other edible fruits, such as strawberry (21.2–41.7 mg cy-3-*O*-glu/100g FW), plum (56.0–124.5 mg/100g FW), red grape (48.0–121.1 mg/100g FW), cherry (32.0–122.0 mg/100g FW), blackberry (100.0–300.5 mg/100g FW) and cranberry (140 mg/100g FW). However, their anthocyanin content is in the range of blackcurrant (476 mg/100g FW), elderberry (1375 mg/100g FW), chokeberry (1480 mg/100g FW) and black raspberry (687 mg/100g FW) [19,28].

Considering a serving size of 150 g of blueberry, the anthocyanin content of the blueberry varieties under study would range from 577.2 to 2494.5 mg cy-3-*O*-glu/serving.

### 3.2. Antioxidant Capacity: ORAC Assay

The antioxidant capacity of the analysed blueberry anthocyanin-rich extracts is presented in Figure 1, expressed as μmol TE/100 g FW. Antioxidant activity followed similar trend to TAC. The Star showed the highest value (6345 ± 601 μmol of TE/100 g FW), 2.8-fold higher than Snowchaser, which presented the lowest (2231 ± 131 μmol of TE/100 g FW). Cristina Blue and Stella Blue (5513 ± 580 and 5251 ± 534 μmol of TE/100 g FW, respectively) displayed intermediate values with no significant differences. 

Kalt et al. [74] determined the antioxidant capacity of 20 blueberry varieties (Bluecrop, Duke, Brigitta, Bluejay, Legacy and Sampson, among others), whose average antioxidant activity was 4900 μmol of TE/100 g FW. Bunea et al. [31] reported ORAC values for Bluegold, Nui, Darrow, Legacy, Nelson, Hanna’s Choice and Toro varieties between 2036–3458 μmol TE/100 g FW, with Hanna’s Choice standing out for having the lowest antioxidant activity, and Toro the highest (Table 2). Wang et al. [73] estimated the ORAC value for 14 different varieties between 2627 and 6747 μmol TE/100 g FW, with Northland standing out as the variety with the highest antioxidant capacity, and Berkley with the lowest (Table 2). The present study showed that Snowchaser’s antioxidant value is within the range previously described for Berkeley, Brie G Kobita, Bluegold, Nui, Darrow and Hanna’s Choice varieties, whereas Star is similar to North Country (Figure 1 and Table 2). Stella Blue and Cristina Blue values agree with the Blomidom, Chipava and Send a Blow varieties.

Star, Cristina Blue and Stella Blue ORAC values were in the range of other edible fruits such as blackberry (6250–8550 μmol TE/100 g FW), honeyberry (5200–6800 μmol TE/100 g FW) and red grape (3700–13,500 μmol TE/100 g FW) [75,76]. Other berries, such as cranberry and elderberry, showed higher ORAC values (7000 and 20,500 μmol TE/100 g FW, respectively) [56].

The antioxidant capacity of the four blueberry anthocyanin-rich extracts was positively correlated with the TAC (*r* = 0.72; *p* < 0.0001), as assessed by the ORAC assay, agreeing with other reports [62,75,77]. This shows that the high antioxidant activity of the extracts can, in fact, be attributed to the TAC. Thus, a subsequent linear regression analysis was performed to evaluate the effect of the TAC in the tested blueberries on their antioxidant activity, obtaining the model in Equation (1):

ORAC = 3006.64 + 2.23 × TAC (3)

The model had an average fit—only *R^2^* = 0.52—showing that about half of the variability in the data is explained by other variables. However, both the model and the TAC model parameters were statistically significant (*p* < 0.0001). Borges et al. [78] reported that the anthocyanins are indeed the major contributor to blueberries’ antioxidant activity (84%). They showed that a broad spectrum of anthocyanins (15 major contributors) are responsible: delphinidin-3-Ogalactoside (≈20%), cyanidin-3-*O*-galactoside and delphinidin-3-*O*-arabinoside (≈15%), petunidin-3-Ogalactoside (≈12%), malvidin-3-*O*-galactoside (≈12%), malvidin-3-*O*-arabinoside (≈9%).

### 3.3. Anthocyanin Profile

A total of 25 different anthocyanin compounds were identified by UHPLC-MS/MS in the four blueberry varieties (Table 3). Star and Stella Blue presented the highest diversity on the anthocyanin profile (22 and 23 different compounds, respectively). Nineteen different anthocyanins were described for Cristina Blue and Snowchaser. The previously described anthocyanin profiles vary between 10–23 different compounds, depending on the variety, with Brigitta having the highest number [21,31,38,41,76,79]. Therefore, the varieties analysed in the present study are among the blueberry varieties with highest diversity of anthocyanin compounds.

Star, Stella blue, Snowchaser and Cristina blue presented five out of the six most frequently naturally occurring anthocyanidins on their profile (cyanidin, delphinidin, peonidin, petunidin and malvidin), showing a great diversity (Table 3). Most of the varieties previously reported present these anthocyanidins, except for Bluegold, Brigitta and Legacy cultivated in Chile; Rocio and the two experimental varieties (V2 and V3) grown in Spain and Bluecrop cultivated in USA, which did not contain peonidin [36,41,80]. Anthocyanins present in the varieties studied were the glucoside, arabinoside, rutinoside, acetyl and malonyl forms (Table 3). Additionally, xyloside, p-coumaroyl and caffeoyl forms had been previously identified in Brigitta and Bluecrop grown in Chile and Macedonia, respectively [31,79]. However, in the hot climate of Spain the acetylated forms of anthocyanins had been found in experimental cultivars, V2 and V3, only [41].

Most of the anthocyanin compounds were present in all four varieties under study (Table 3). However, cyanidin-3-rutinoside, petunidin-3-rutinoside and malvidin-3-(6″-acetyl) galactoside were only identified in Stella blue (Table 3). As far as we know, this is the first time that cyanidin-3-rutinoside has been identified in blueberry. It could be proposed as a marker of the Stella Blue variety. This compound had been previously identified in strawberry, blackberry, sweet berry, Chilean guava (Ugni molinae) and plum [21,23,81]. Petunidin-3-rutinoside was previously described in Brigitta blueberry variety, while malvidin-3-(6″-acetyl) galactoside was also identified in the Arlen, Legacy, Lenoir, O’Neal, Pamlico, Sampson, Toro and Bluecrop varieties [21,38,79].

Delphinidin-3-(6″-acetyl) glucoside was only identified in Star (Table 3), although it had previously been detected in varieties, such as Brigitta, Arlen, Legacy, Lenoir, O’Neal, Pamlico, Sampson, Bluecrop, and Ozarkblue [21,38,76]. Cyanidin-3-(6″-acetyl) galactoside was detected in Star and Cristina Blue (Table 3). As far as we know, this compound had only been detected previously in a non-declared blueberry variety cultivated in USA [81]. Petunidin-3-(6″-acetyl) galactoside was identified in Star, Snowchaser and Stella Blue (Table 3), and it was only previously reported in a non-declared blueberry variety grown in Slovenia [82]. Similarly, delphinidin-3-(6″-malonyl) glucoside was also determined in Star, Snowchaser and Stella Blue (Table 3). Conversely, it was only reported in the abovementioned non-declared blueberry variety cultivated in USA [81]. Although peonidin-3-*O*-glucoside and peonidin-3-*O*-rutinoside were identified in the four varieties studied, they were only reported in the abovementioned blueberry varieties grown in USA and Slovenia, and in Brigitta, respectively [81,82]. Peonidin glycosides usually reported in blueberries are galactoside and arabinoside [21,31,38,76,79,81,83]. Therefore, we could propose Cyanidin-3-(6″-acetyl) galactoside as a marker for Star and Cristina Blue; petunidin-3-(6″-acetyl) galactoside and delphinidin-3-(6″-malonyl) glucoside as markers for Star, Snowchaser and Stella Blue, and peonidin-3-*O*-glucoside and peonidin-3-*O*-rutinoside markers for Star, Snowchaser, Cristina Blue and Stella Blue, including the previously-reported Brigitta for the latter compound.

### 3.4. Antibacterial Activity 

Table 4 presents the minimum inhibitory concentration (MIC) of the anthocyanin-rich extracts from the four varieties of blueberries against the strains isolated from urinary tract infections (UTI) and standard strains. All varieties significantly inhibited the growth of the eight strains (five UTI strains and three standard strains) when compared with the negative control (70% methanol), with MICs ranging from 0.4 mg/mL (in the case of Stella Blue extract against UTI *P. aeruginosa*) to 9.5 mg/mL (in the case of all extracts against UTI *K. pneumoniae* ssp. *pneumoniae*) (Table 4).

Our results show that the 4 anthocyanin-rich extracts were most effective against *P. aeruginosa* among the bacteria isolated from UTI patients. This is surprising, because *P. aeruginosa* was the strain most resistant to the positive control general wide-spectrum antibiotic Gentamicin. This finding suggests the need to further explore the potential of anthocyanins from blueberries as a novel approach to controlling some antibiotic resistant UTI strains, especially since we are currently facing the problem of the emergence and spread of antibiotic-resistant strains. UTI can, however, be caused by many pathogens, with uropathogenic *E. coli* as the main culprit identified in about 80% of the clinical cases [84]. In this respect, the four anthocyanin-rich extracts were less effective against *K. pneumoniae* UTI (the most resistant strain), followed by *E. coli* UTI and ATCC strains. These results were later confirmed by minimum bactericidal concentration (MBC) testing (Table 5). 

The MBC ranged from 1.0 mg/mL (in the case of extracts against UTI *P. aeruginosa* and Stella Blue against UTI *Micrococcus* spp.) to 9.5 mg/mL (in the case of all extracts against UTI *K. pneumoniae* ssp. *pneumoniae*, Star against UTI *E. coli*; Stella Blue against ATCC *E. coli* and Star against *L. monocytogenes*) (Table 5). The blueberry anthocyanin-rich extracts had a statistically similar effect to the synthetic generic antibiotic (0.4 mg/mL Gentamicin) used as positive control against all five UTI strains tested, and only against *E. coli* from the standard ATCC strains. The effect was weaker than the positive control against *S.* Enteritidis and *L. monocytogenes*, but significant, nonetheless. Hence, the extracts were the most effective against *P. aeruginosa* (MBC = 1.0 mg/mL), while the smallest effect was against *K. pneumoniae* (MBC = 9.5 mg/mL). Similarly, *K. pneumoniae* showed to be resistant to anthocyanins-rich pomegranate extracts [85].

The effect of the four anthocyanin-rich extracts against the UTI strains tested as a group was compared with the ATCC strains (data not shown). The results suggested that each of the four extracts are approximately 3-fold less effective against UTI Gram-negatives than against ATCC Gram-negatives. The same remark could be made when considering the anthocyanin-rich extracts as one group only, supporting all the observations made within this study. However, this can be seen only in the case of Cristina Blue for Gram-positives, and not at all for anthocyanin-rich extracts as a whole. The differences were also evaluated between the tested Gram-negatives and Gram-positives. The effect of the four blueberry varieties did not vary between the two groups. However, the extracts were 3-fold less effective against the UTI than the ATCC within the Gram-negatives group, consistent with the prior observation. This observation is in line with the reports of Dorneanu et al. [59] that show a greater resistance of Gram-negative UTI strains to *Aronia melanocarpa* anthocyanin extracts compared to Gram-positives. Even though the Gram-negative UTI strains appear to be consistently more resistant to the anthocyanins from blueberries, the main limitation of this study is that only one Gram-positive strain was isolated from the UTI compared to four Gram-negatives. Thus, the data on Gram-positives is rather limited. This could open future research paradigms for the antibacterial activity of anthocyanins.

Table 6 shows the Pearson correlation between the antibacterial activity of the four anthocyanin-rich extracts and the TAC and ORAC. Although positive correlations between the MIC, MBC and the TAC were obtained, it reached statistical significance in the case of the UTI β-Haemolytic *E. coli* MBC only. Interestingly, the MIC of *S.* Enteritidis showed an inverse relation with the TAC; although high, it was not significant. No correlation was observed between the antibacterial and antioxidant activity. This agrees with other studies [55], although other authors have found a significant correlation between antibacterial activity and polyphenolic content in berries [32]. Linear regression analysis was performed following the results of the Pearson correlation. The model equations and the goodness of fit parameters are presented in Table 7.

The mechanism of bacterial inhibition by berry compounds is an accumulation of direct and indirect effects [86]. The direct action is the interference of phytochemicals with the bacterial cell membrane that leads to the inactivation of crucial enzymes. The indirect effect, on the other hand, is related to the nutrient availability or genomic expression, both impairing the metabolism and the normal functioning of the bacteria. Blueberry anthocyanins act mainly by inhibiting gene transcription, disrupting the cell membrane structure and energy transport, thus inhibiting their growth and reproduction [51,52,60,87]. Blueberry extracts were observed to affect the transcription of up to seven genes in the bacterial cell [51,60]. These genes had critical roles in the internal and external channels of cell membranes. Among these was the TolC porin protein that controls the outer membrane channel. It also has a central role in pumping out antibacterial agents such as antibiotics and detergents. Other studies showed that blueberry anthocyanins distorted the membrane morphology and caused aggregation and leakage of cellular contents of *E. coli*, *S.* Typhimurium, *S.* Enteritidis, *L. monocytogenes* and *P. aeruginosa* [46,52,60,87]. The anthocyanins from blueberries might also interfere with the activity of several enzymes regulating the bacterial cell’s metabolic functions [45,52]. They could inactivate the alkaline phosphatase (AKP), thus preventing cell differentiation, and they may affect the Ca^2+^ metabolism as well. Additionally, blueberry anthocyanins might lower the levels of ATPase, increasing the efflux of ATP from the cytoplasm of pathogens, thus inhibiting respiratory metabolism and affecting the energy supply. 

Furthermore, sublethal concentrations of blueberry pomace extracts significantly affected other factors related to the virulence and pathogenicity of pathogens. They decreased the cell surface hydrophobicity of *S.* Typhimurium, together with its auto-aggregation, cellular motility, colonisation and invasion capabilities [87]. Similarly, compounds in purified proanthocyanins from cranberry extracts inhibited the agglutination of *E. coli* and *K. pneumoniae* [88]. However, this effect was strain- specific and dose-dependent [53,88]. Blueberry extracts affected the growth of *E. coli* and *P. aeruginosa*, and they were able to significantly inhibit their biofilm formation and bacterial adhesion [50]—both important factors in their surface colonisation and infection [54]. Low concentrations of blueberry extracts proved to be more effective in inhibiting the biofilm formation, because a higher anthocyanin concentration could increase the production of exopolysaccharides in the presence of environmental stress, enhancing the protection of bacteria [50]. The specific mechanism of blueberry anthocyanins needs, therefore, to be further explored and qualified. Similar significant results were reported for anthocyanins from blueberries in general against *E. coli* [47,50,89] and *L. monocytogenes* foodborne pathogens [47,52], and for UTI pathogens *E. coli* [50,57,58] and *P. aeruginosa* [50].

Comparing our results with others already reported, a lower inhibition of blueberry anthocyanins was observed for *E. coli* O157:H7 (MIC = 173.08 mg/mL) [46]; of various blueberry cultivars (Highbush and Rabbiteye) extracts for *E. coli* (MIC = 20–35 mg/mL) [32] and of blueberry fruit infusion for *E. coli* (MIC = 50 mg/mL) [89]. Additionally, Sun et al. [52] obtained lower MICs and MBCs of *L. monocytogenes* and *S.* Enteritidis (MIC=0.27 mg/mL; MBC = 0.53) for blueberry anthocyanins. Other studies dealing with blueberry extracts presented MIC of *L. monocytogenes* ranging from 100 to 300 mg/mL and an MBC of 100 to 450 mg/mL, while for *S.* Enteritidis, a MIC of 100 to 450 mg/mL and an MBC of 100 to 600 mg/mL [49,53]. However, Zhou et al. [53] also suggested that *L. monocytogenes* might be more resistant than *S.* Enteritidis. Other studies, on the contrary, showed that blueberry peel extracts were most effective against Gram-negative bacteria, and mainly against *E. coli* [90]. Zhou et al. [53] also observed a higher inhibition of Gram-negative *S.* Enteritidis compared to the Gram-positive *L. monocytogenes* by blueberry extracts, including anthocyanins and proanthocyanidins. This effect, however, was not observed in the present study. 

Not only, therefore, do the results vary, depending how the berries are processed prior to extraction and the type of extraction solvent used [86,91], but also on their variety and the pedo-climatic conditions in which they were cultivated [44,47,48,49,53,86]. Additionally, many phenolic compounds from blueberries, anthocyanins included, do not only directly inhibit the growth and survival of bacteria, but they do affect their virulence factor and their antibiotic resistance [47,51,52]. This makes their antibacterial activity also dependent on the strain tested.

Both cranberries and blueberries share a similar anthocyanin profile [31,44,91,92]. It appears that the monoglycoside anthocyanins (such as: delphinidin-3-*O*-glucoside, petunidin-3-*O*-glucoside, cyanidin-3-*O*-glucoside, malvidin-3-*O*-glucoside, peonidin-3-*O*-glucoside), abundant in blueberries, might be the compounds with the highest antibacterial effect against *S.* Enteritidis and *L. monocytogenes* [44,48]. A mixture of anthocyanins may therefore be needed in order to inhibit pathogens successfully [60,61,62]. In addition, the anthocyanins may exert an antibacterial activity, because they have the double ability to donate protons, causing the hyper acidification of the plasma membrane, and to sequester electrons from the respiration process [47].

Blueberry extracts showed better antibacterial activities than those of raspberry and strawberry, but were generally lower than cranberry extracts [45]. However, anthocyanins and proanthocyanidins from black chokeberries and pure cyanidin-3-O-galactoside proved to be ineffective against standard strains of *E. coli*, *S. enterica*, *L. monocytogenes* nor *P. aeruginosa* [55]. This shows the importance of not only the berries’ anthocyanin profile, but also of having a broad spectrum of active compounds that could act synergistically [61,62]. Results highly comparable to the present study were obtained in a parallel study on the effect of *Aronia melanocarpa* on a broad spectrum of UTI clinical isolates strains, including *E. coli*, *P. aeruginosa* and *K. pneumoniae* [59]. The MIC for the strains of interest ranged from 2.5 mg/mL to 10 mg/mL.

The role of anthocyanins from berries (mostly cranberries and blueberries) in UTI prevention and/or treatment still remains unclear. They interfere in vitro with the most prevalent and important virulence factor—the adhesion—of uropathogenic *E. coli* [46,50,58]. To the best of our knowledge, there are no in vivo studies relevant to the antibacterial activity of anthocyanins from blueberries. However, Ibrahim et al. [93] showed that cranberry anthocyanin extracts (200 mg/kg b.w aqueous and methanol extracts) were effective in treating UTI caused by *E. coli* O157:H7 in infected rats. Although the anthocyanin profiles are not exactly the same in blueberries and cranberries, we might expect a similar behaviour. Nevertheless, further in vitro and in vivo studies would be needed, to ascertain not only the inactivation mechanism, but also that the in vitro effects can be transferred to in vivo.

It appears that the concentration and type of anthocyanins reaching the urine of UTI patients might be insufficient to exert a quantifiable effect [88]. In this sense, Cochrane meta-analyses, including clinical studies, showed a lack of positive trials [16,18], while the Spanish Urological Association concluded that there are no significant benefits compared with placebo, except for a very small effect in certain population subgroups [94]. However, the positive results in this and other in vitro studies [50,57,58] seem to point out that the actual optimal extraction procedures, mixture formulations, dosage and bioavailability are not yet clear [88]. Moreover, anthocyanins in blueberries might target each individual uropathogenic strain in the wider spectrum of UTI pathogens differently, varying their response to treatment [88]. It becomes, therefore, important first to verify that the correct bacteria are targeted before randomised clinical testing, an objective addressed by this study. However, based on in vitro data alone no recommendation for clinical practice could possibly be formulated. Further studies are needed to understand properly the pharmacokinetics of anthocyanins from blueberries and to establish properly the correct doses, in order to achieve a preventive and/or therapeutic concentration in the urinary tract [88].

## 4. Conclusions

Our results show that Star, Cristina Blue and Stella Blue blueberry varieties cultivated in the hot climate of Southern Spain exhibited significantly higher TAC values than the richest variety in anthocyanins so far reported. Star showed the highest antioxidant activity value, followed by Cristina Blue, Stella Blue and Snowchaser. As far as we know, this is the first time that cyanidin-3-rutinoside has been identified in blueberry. We could propose cyanidin-3-(6″-acetyl) galactoside as a marker for Star and Cristina Blue; petunidin-3-(6″-acetyl) galactoside and delphinidin-3-(6″-malonyl) glucoside as markers for Star, Snowchaser and Stella Blue; and peonidin-3-*O*-glucoside and peonidin-3-*O*-rutinoside markers for Star, Snowchaser, Cristina Blue and Stella Blue, including the previously-reported Brigitta for the latter compound. The anthocyanin-rich extracts from the four varieties effectively inhibited all the tested UTI and standard strains, with MICs ranging from 0.4 mg/mL (in the case of Stella Blue extract against UTI *P. aeruginosa*) to 9.5 mg/mL (in the case of all extracts against UTI *K. pneumoniae* ssp. *pneumoniae*). They were surprisingly effective against the *P. aeruginosa* UTI strain, showing a possible new approach in the endeavour to seek new measures for controlling some antibiotic resistant UTI strains. To the best of our knowledge, this is the first study that has tested in vitro the antibacterial activity of blueberries against *Klebsiella pneumoniae*, *Providencia stuartii* and *Micrococcus* spp. strains isolated from UTI. Therefore, we have shown that the principal potential uropathogenic bacteria are in fact targeted by the anthocyanin-rich extracts from blueberries. Our results offered a first tentative insight into the potential spectrum of UTI pathogens affected by the anthocyanins in blueberries, highlighting the strain-specificity of the antibacterial effect.

## Figures and Tables

**Table 1:** Ratio of anthocyanin extract obtained from each blueberry variety.

Variety	Ratio (mg Anthocyanin Extract/100 g FW)
Star	704.9
Snowchaser	253.8
Cristina Blue	431.7
Stella Blue	439.1

**Table 2:** Antioxidant activity of different blueberry extracts (*V. corymbosum*).

Variety	ORAC (µmol TE/100g FW)	Cropfile	References
Blomidom	5538 ± 388	USA	[73]
Northland	6747 ± 121	USA	[73]
Northblue	4976 ± 722	USA	[73]
North Country	6265 ± 699	USA	[73]
Chipava	5856 ±165	USA	[73]
Elliott	4098 ± 436	USA	[73]
Darrow	3994 ± 522	USA	[73]
Bluecrop	4491 ± 190	USA	[73]
Primary Operation Blue	3649 ± 473	USA	[73]
Send a Blow	5070 ± 179	USA	[73]
Berkley	2627 ± 364	USA	[73]
JK-M7	4114 ± 344	USA	[73]
Brie G Kobita	2737 ± 394	USA	[73]
Duke	3145 ± 263	USA	[73]
Bluegold	2121 ± 326	Romania	[31]
Nui	2235 ± 677	Romania	[31]
Darrow	2543 ± 219	Romania	[31]
Legacy	2899 ± 531	Romania	[31]
Nelson	3027 ± 474	Romania	[31]
Hanna’s Choice	2036 ± 223	Romania	[31]
Toro	3458 ± 325	Romania	[31]

**Table 3:** Tentative identification of different anthocyanins in the blueberry varieties analysed.

Retention Time (min)	Compounds	Molecular Formula (M^+^)	Calculated Mass (*m/z*)	Accurate Mass (*m/z*)	Error (ppm)	MS/MS Fragments	Variety
4.03	Delphinidin-3-*O*-galactoside	C_21_H_21_O_12_	465.1028	465.1023	−1.0441	303.0501	A; B; C; D
4.30	Delphinidin-3-*O*-glucoside *	C_21_H_21_O_12_	465.1028	465.1025	−0.4535	303.0502	A; B; C; D
4.44	Cyanidin-3-galactoside *	C_21_H_21_O_11_	449.1078	449.1073	−1.1349	287.0552	A; B; C; D
4.47	Delphinidin-3-*O*-arabinoside	C_20_H_19_O_11_	435.0922	435.0918	−0.8334	303.0501	A; B; C; D
4.60	Cyanidin-3-*O*-glucoside *	C_21_H_21_O_11_	449.1078	449.1073	−1.1349	287.0543	A; B; C; D
4.65	Petunidin-3-*O*-galactoside	C_22_H_23_O_12_	479.1184	479.1180	−0.9383	317.0658	A; B; C; D
4.68	Cyanidin-3-*O*-rutinoside	C_27_H_31_O_15_	595.1658	595.1650	−1.1945	449.10784/ 287.05501	D
4.73	Cyanidin-3-*O*-arabinoside	C_20_H_19_O_10_	419.0973	419.0968	−1.1173	287.0550	A; B; C; D
4.76	Petunidin-3-*O*-glucoside	C_22_H_23_O_12_	479.1184	479.1179	−1.1346	317.0645	A; B; C; D
7.77	Petunidin-3-*O*-rutinoside	C_28_H_33_O_16_	625.1763	625.1766	0.4215	479.1180	D
4.89	Peonidin-3-*O*-galactoside	C_22_H_23_O_11_	463.1235	463.1230	−1.1500	301.0708	A; B; C; D
4.89	Petunidin-3-*O*-arabinoside	C_21_H_21_O_11_	449.1078	449.1073	−1.1300	317.0656	A; B; C; D
4.99	Malvidin-3-*O*-galactoside	C_23_H_25_O_12_	493.1341	493.1335	−1.0913	331.0812	A; B; C; D
4.99	Peonidin-3-*O*-glucoside *	C_22_H_23_O_11_	463.1235	463.1231	−0.7546	301.0694	A; B; C; D
5.04	Peonidin-3-*O*-rutinoside	C_28_H_33_O_15_	609.1814	609.1804	−1.7904	301.0691	A; B; C; D
5.10	Malvidin-3-*O*-glucoside *	C_23_H_25_O_12_	493.1341	493.1336	−0.8437	331.0802	A; B; C; D
5.19	Cyanidin-3-(6″-acetyl) galactoside	C_23_H_23_O_12_	491.1184	491.1181	−0.6047	287.0551	A; C
5.22	Malvidin-3-*O*-arabinoside	C_22_H_23_O_11_	463.1235	463.1231	−0.9568	331.0813	A; B; C; D
5.33	Delphinidin-3-(6″-acetyl) glucoside	C_23_H_23_O_13_	507.1133	507.1133	−0.7986	303.0497	A
5.33	Petunidin-3-(6″-acetyl) galactoside	C_24_H_25_O_13_	521.1290	521.1236	−1.1710	317.0657	A; B; D
5.39	Cyanidin-3-(6-acetyl) glucoside	C_23_H_23_O_12_	491.1184	491.1182	−0.3561	287.0551	A; C; D
5.47	Malvidin-3-(6″-acetyl) galactoside	C_25_H_27_O_13_	535.1145	535.1441	−0.9590	331.0801	D
5.48	Petunidin-3-(6″-acetyl) glucoside	C_24_H_25_O_13_	521.1290	521.1289	−0.2340	317.0651	A; B; C; D
5.53	Peonidin-3-(6″-acetyl) glucoside	C2_4_H_25_O_12_	505.1341	505.1343	0.4501	301.0707	A; B; C; D
5.58	Delphinidin-3-(6″-malonyl) glucoside	C_24_H_23_O_15_	551.1032	551.1034	0.4419	303.0501	A; B; D

* Identified with pure standards; A: Star; B: Snowchaser; C: Cristina Blue and D: Stella Blue.

**Table 4:** Minimum inhibitory concentration (MIC) of anthocyanin-rich extracts from the four varieties of blueberries, expressed in mg/mL.

Type of Strain		UTI Strains					ATCC Standard Strains		
Gram staining of the strains		Gram-negative				Gram-positive	Gram-negative		Gram-positive
	**Strains**	*Klebsiella pneumoniae* ssp. *pneumoniae*	*Providencia stuartii*	*Escherichia coli* β-Haemolytic	*Pseudomonas aeruginosa*	*Micrococcus* spp.	*Escherichia coli* ATCC 25922	*Salmonella* Enteritidis ATCC 13076	*Listeria monocytogenes* ATCC 19114
**Sample**									
Cristina Blue		9.52 ± 0 ^Ac^	1.78 ± 0.65 ^A,a^	3.74 ± 1.372 ^A,b^	0.85 ± 0.31 ^A,a^	1.41 ± 0.65 ^A,a^	3.74 ± 1.37 ^A,b^	2.16 ± 0 ^C,a^	4.54 ± 0 ^D,b^
Star		9.52 ± 0 ^Ad^	1.78 ± 0.65 ^A,a,b^	3.74 ± 1.372 ^A,c^	0.49 ± 0 ^A,a^	2.57 ± 1.79 ^A,b,c^	3.74 ± 1.37 ^A,c^	1.03 ± 0 ^B,a,b^	2.16 ± 0 ^C,a,b,c^
Snowchaser		9.52 ± 0 ^Ad^	1.78 ± 0.65 ^A,a,b^	3.74 ± 1.372 ^A,c^	0.58 ± 0.41 ^A,a^	1.78 ± 0.65 ^A,a,b^	3.74 ± 1.37 ^A,c^	1.78 ± 0.65 ^C,a,b^	2.16 ± 0 ^C,b^
Stella Blue		9.52 ± 0 ^Ad^	1.78 ± 0.65 ^A,a,b^	3.74 ± 1.372 ^A,c^	0.40 ± 0.15 ^A,a^	0.76 ± 0.46 ^A,a,b^	3.74 ± 1.37 ^A,c^	2.16 ± 0 ^C,b^	1.41 ± 0.653 ^B,a,b^
Positive control (µg/mL)		0.28 ± 0.20^Aa^	1.45 ± 0.67 ^A,b^	0.12 ± 0.11 ^A,a^	3.85 ± 1.41 ^A,c^	0.02 ± 0 ^A,a^	0.05 ± 0.05 ^A,a^	0.69 ± 0.32 ^A,a,b^	0.02±0 ^A,b^

Note: The data are presented as mean ± SD, *n* = 3. Different uppercase letters indicate statistically significant differences on the columns, and therefore differences among the samples, while the different lowercase indicate statistically significant differences on the rows, therefore differences among the different strains (Fisher LSD, *p* < 0.05). The negative control was 70% methanol, the solvent used for the dilution of the lyophilised anthocyanins, while the positive control was Gentamicin (with the initial concentration of stock solution of 0.4 mg/mL).

**Table 5:** Minimum bactericidal concentration (MBC) of anthocyanin-rich extracts from the four varieties of blueberries, expressed in mg/mL.

Type of Strain		UTI Strains					ATCC Standard Strains		
Gram staining of the strains		Gram-negative				Gram-positive	Gram-negative		Gram-positive
	**Strains**	*Klebsiella pneumoniae* ssp. *pneumoniae*	*Providencia stuartii*	*Escherichia coli* β-Haemolytic	*Pseudomonas aeruginosa*	*Micrococcus* spp.	*Escherichia coli* ATCC 25922	*Salmonella* Enteritidis ATCC 13076	*Listeria monocytogenes* ATCC 19114
**Sample**									
Cristina Blue		9.52	4.54	4.54	1.03	2.16	4.54	2.16	4.54
Star		9.52	4.54	9.52	1.03	4.54	4.54	4.54	9.52
Snowchaser		9.52	2.16	4.54	1.03	2.16	4.54	4.54	4.54
Stella Blue		9.52	2.16	4.54	1.03	1.03	9.52	2.16	2.16

**Table 6:** Pearson correlation matrix of the minimum inhibitory concentration (MIC) and minimum bactericidal concentration (MBC), with the total anthocyanin content (TAC) of the four varieties of blueberries and the antioxidant capacity, as assessed by the ORAC.

Type of strain		UTI Strains					ATCC Standard Strains		
**Gram staining of the strains**		Gram-negative				Gram-positive	Gram-negative		Gram-positive
	**Strains**	*Klebsiella pneumoniae* ssp. *pneumoniae*	*Providencia stuartii*	*Escherichia coli* β-Haemolytic	*Pseudomonas aeruginosa*	*Micrococcus* spp.	*Escherichia coli* ATCC 25922	*Salmonella* Enteritidis ATCC 13076	*Listeria monocytogenes* ATCC 19114
**Sample**									
TAC		0.69	−0.24	−0.81	−0.08	0.86	0.69	**0.96**	−0.22
ORAC		0.13	−0.04	−0.25	0.18	0.39	0.70	0.56	0.15

Values in bold are different to 0 with a significance level of α = 0.05.

**Table 7:** Regression of antibacterial activity (MIC and MBC) variables and the total anthocyanin content (TAC) of the four varieties of blueberries, and the antioxidant capacity, as assessed by the ORAC.

Linear Regression Equations	Goodness of Fit, *R^2^*	Statistical Significance of the Model, *p*
MIC S. ATCC = 1.74 − 1.46 × 10^−3^ × TAC + 2.69 × 10^−4^ × ORAC	1.00	0.002
MBC E. UTI = 3.64 + 5.83 × 10^−3^ × TAC − 6.00 × 10^−4^ × ORAC	0.99	0.023
MBC S. ATCC = 5.64 + 3.62 × 10^−3^ − 1.12 × 10^−3^ × ORAC	0.99	0.033
MBC E. UTI = 2.03 + 4.33 × 10^−3^ × TAC	0.92	0.039

Where: The first letter in the abbreviation stands for the variable correlated: MIC—minimum inhibitory concentration; MBC = minimum bactericidal concentration; the letters afterwards show the strain tested: E.—*Escherichia coli*; S.—*Salmonella* Enteritidis; while the type of isolation is last: UTI—strain isolated from urinary tract infection; ATCC—standard strain.
